# VBCNet: A Hybird Network for Human Activity Recognition

**DOI:** 10.3390/s24237793

**Published:** 2024-12-05

**Authors:** Fei Ge, Zhenyang Dai, Zhimin Yang, Fei Wu, Liansheng Tan

**Affiliations:** School of Computer Science, Central China Normal University, Wuhan 430070, China; zhenyangdai@mails.ccnu.edu.cn (Z.D.); zhiminyang@mails.ccnu.edu.cn (Z.Y.); wufei0522@mails.ccnu.edu.cn (F.W.); l.tan@mail.ccnu.edu.cn (L.T.)

**Keywords:** Wi-Fi channel state information, body-coordinate velocity profile, ViT, BiLSTM, convolutional feed-forward

## Abstract

In recent years, the research on human activity recognition based on channel state information (CSI) of Wi-Fi has gradually attracted much attention in order to avoid the deployment of additional devices and reduce the risk of personal privacy leakage. In this paper, we propose a hybrid network architecture, named VBCNet, that can effectively identify human activity postures. Firstly, we extract CSI sequences from each antenna of Wi-Fi signals, and the data are preprocessed and tokenised. Then, in the encoder part of the model, we introduce a layer of long short-term memory network to further extract the temporal features in the sequences and enhance the ability of the model to capture the temporal information. Meanwhile, VBCNet employs a convolutional feed-forward network instead of the traditional feed-forward network to enhance the model’s ability to process local and multi-scale features. Finally, the model classifies the extracted features into human behaviours through a classification layer. To validate the effectiveness of VBCNet, we conducted experimental evaluations on the classical human activity recognition datasets UT-HAR and Widar3.0 and achieved an accuracy of 98.65% and 77.92%. These results show that VBCNet exhibits extremely high effectiveness and robustness in human activity recognition tasks in complex scenarios.

## 1. Introduction

In this era of rapid development of the Internet of Things (IoT), human–computer interaction has become a core component of this technological landscape. Devices such as cameras [1], mobile phones, and sonar [2] have facilitated diverse and effective interaction methods. However, in the context of continuous technological advancement, there is a growing concern about personal privacy issues, such as unauthorised use of portraits or illegal synthesis of voices [3]. Therefore, there is an urgent need for a method that enables human–computer interaction while protecting privacy (especially portraits and voices). In this context, the use of Wi-Fi signals for human gesture recognition is particularly appropriate. Currently, Wi-Fi has been widely deployed in homes, schools, business centres, etc., which effectively avoids the need for large-scale installation of additional equipment, and thanks to the penetrating nature of Wi-Fi signals, it does not need to interact directly with the user face-to-face, and thus can protect the user’s privacy to a large extent [4] and reduce the user’s concern about privacy leakage.

Wi-Fi signals convey key information such as Channel State Information (CSI) and Received Signal Strength Indication (RSSI). RSSI, primarily influenced by multipath fading in indoor environments, is commonly used to estimate the distance of moving objects and serves as a foundation for indoor positioning systems [5,6,7]. On the other hand, CSI provides detailed insights into wireless signals by leveraging the Orthogonal Frequency Division Multiplexing (OFDM) technique. It captures amplitude and phase variations across different subcarriers, reflecting the physical phenomena (e.g., reflection, diffraction, and attenuation) encountered during signal propagation. For instance, human activities like falls, gestures, or limb movements disrupt the propagation paths of wireless signals, resulting in changes to the CSI. Due to its ability to capture these fine-grained environmental changes, CSI demonstrates superior reliability and accuracy in human behaviour recognition tasks compared to RSSI [8,9,10,11,12].

In early studies, researchers attempted to achieve the classification task by manually extracting features from channel state information (CSI) data [8]. However, this manual feature extraction method is difficult to fully capture the unique features of different activity types, and its efficiency and accuracy are limited. With the rapid development and maturity of deep learning technologies, researchers have gradually recognised that the performance of human activity recognition (HAR) tasks can be greatly enhanced by utilising the powerful feature learning capabilities of deep learning models such as MLP [13], CNN [14], and CNN-LSTM [15], which are able to automatically learn rich and hierarchical feature representations from complex CSI data.

CNNs excel in extracting local complex features of CSI signals, but face challenges in capturing global feature correlations. For this reason, researchers have proposed some innovative models. For example, ref. [16] proposes an innovative model called space-temporal convolution with nested long short-term memory (STC-NLSTMNet), a novel architecture that extracts spatial and temporal features by combining spatio-temporal convolution with nested long short-term memory network (NLSTM). And another study [17] combines the channel attention mechanism with the CNN-LSTM model, which dynamically adjusts the channel feature weights on the basis of extracting temporal and spatial features and assigns higher weights to the key channels. These methods have achieved better results in integrating local features and time dependence, but are still insufficient in capturing long-distance dependence and global features.

In 2020, Google Research proposed Vision Transformer (ViT) [18] based on Transformer [19]. Thanks to Transformer’s self-attention mechanism that can naturally capture long-distance dependencies between different regions in an image, ViT handles the image at the global level in complex relationships, especially excelling in high-dimensional feature spaces. Recently, some researchers have applied ViT to CSI-based human action recognition (HAR), e.g., Zhou et al. [20] proposed a WPFormer architecture that maps the channel state information of Wi-Fi signals to human pose feature points, and then effectively explores the spatial information of human poses through self-attention. Yang [21] proposed WiTransformer for gesture recognition on body-coordinate velocity profile (BVP) data. However, the effective learning of Transformer usually relies on large-scale training data, and the current CSI sequence data are not as rich as image data, so the ability of ViT to extract local features under limited data is not as strong as that of CNN through convolutional operation.

To address these challenges and harness the strengths of different models, this paper introduces a hybrid deep learning framework, VBCNet. In particular, the Encoder layer of VBCNet incorporates a long short-term memory (LSTM) network, which significantly enhances the recognition of continuous actions by effectively capturing the temporal dynamics of CSI sequences. This design improves the model’s ability to identify implicit time-dependent features, especially in modelling long-duration behavioural sequences. Furthermore, to mitigate the limitations of ViT in extracting local features from limited data, VBCNet integrates a convolutional feed-forward (CF) module. By leveraging convolutional operations, the CF module efficiently captures multi-scale and fine-grained features, thereby improving the model’s perception of local information and ensuring robust recognition in diverse environments.

In order to verify the effectiveness of the method in this paper, we conducted extensive experiments on the datasets UT-HAR [22] and Widar3.0 [23]. The experimental results show that the present method achieves 98.56% accuracy on the UT-HAR dataset and excellent results on Widar3.0, which verifies the efficiency and reliability of the hybrid deep learning model based on magnitude information proposed in this study in the human behaviour recognition task.

The main contributions of this paper are as follows:

- We propose a hybrid deep learning-based model, VBCNet, which combines the advantages of Vision Transformer in deep feature extraction with the power of BiLSTM in time series analysis to achieve efficient behavioural classification by extracting high-dimensional feature vectors and transforming them into time series features.
- In order to enhance the ability of ViT to acquire local and multi-scale information, we designed a convolutional feed-forward (CF) module to replace the feedforward network.
- Experiments are conducted on the UT-HAR dataset and the Widar3.0 dataset. By comparing the results with those of several models, such as LSTM, ABLSTM, and CNN-BiLSTM, we demonstrate the performance of VBCNet in subtle human behaviour recognition, showing superior performance.

The rest of the paper is organised as follows: Section 2 describes the form of CSI data collection and the research-related work of HAR in recent years. Section 3 describes how we process the collected CSI data. In addition, the structure of our VBCNet network is described in detail. Section 4 conducts experiments and analyses the results. Finally, conclusions and future work are discussed in Section 5.

## 2. Related Work

This section first introduces the data form of CSI and then describes the progress of human activity recognition research through Wi-Fi signals using deep learning in recent years.

### 2.1. CSI Data

**Figure 1:**
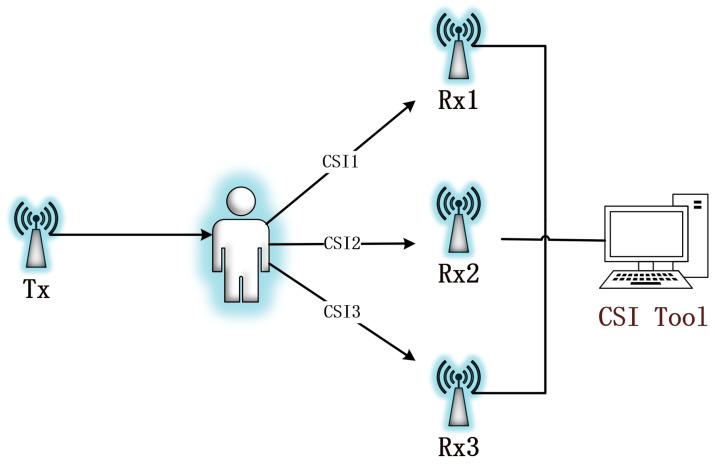
CSI data collection.

Channel state information (CSI) is a key parameter that finely portrays the channel properties of a communication link [24]. By using Orthogonal Frequency Division Multiplexing (OFDM) [25], we are able to extract CSI from the wireless signal between the transmitter and receiver, and Wi-Fi CSI data collection is generally shown in Figure 1.

As fine-grained information in the physical layer, CSI details the state of each channel during wireless communication and can be represented as:

(1) Y=HX+N,

where X represents the signal at the transmitter, N denotes the noise signal in the system, Y is the signal at the receiver, and H is the set of CSI matrices. The H can be represented as:

(2) $$H=\left[\begin{array}{cc} \begin{array}{cc} H_{1,1} & H_{1,2}\\ H_{2,1} & H_{2,2} \end{array} & \begin{array}{cc} \cdots & H_{1,n}\\ \cdots & H_{2,n} \end{array}\\ \begin{array}{cc} H_{3,1} & H_{3,2} \end{array} & \begin{array}{cc} \cdots & H_{3,n} \end{array} \end{array}\right],$$

where n represents the number of subcarriers. The dataset we use originates from a system consisting of a single transmitting antenna and three receiving antennas, with each channel containing 30 subcarriers. Therefore, the amount of data in each packet is 1 × 3 × 30, resulting in a total of 90 data points per packet. Each data point is in complex form, specifically represented as:

(3) $$H_{i,j}=\left|H_{i,j}e^{\varphi_{i,j}}\right|,$$

where $H_{i,j}$ and $\varphi_{i,j}$ represent the amplitude information and phase information, respectively. Here, i represents the antenna index, where i=1,2,3, and j represents the subcarrier index, where j=1,2,⋯,30.

Xiao et al. [26] used CSI amplitude data as parameter input to determine whether there is any human activity by analysing the relationship between the eigenvalues extracted from CSI and the threshold value, while Yang [27] used the difference in signal strength (RSS) between the 2.4 GHz and 5 GHz bands for human location detection. In addition, in order to obtain more stable signals, Yang et al. [28] proposed a CSI-IP method to construct and fuse CSI amplitude and phase images, and Mao et al. [29] further proposed a dual-stream network structure, which is capable of handling variable-length data and processing amplitude in the time domain and phase in the frequency domain, respectively, in order to generate a more comprehensive representation of people’s features. Although these studies have achieved significant results, in order to further reduce the noise impact, Zhou et al. [30] proposed an attention-directed denoising (ADN) method based on CSI-former. This method extracts pose features more efficiently and attenuates noise interference by focusing the network’s attention on subcarriers that are more sensitive to pose while ignoring subcarriers with fewer features and more noise. Considering the objectives and data characteristics of this study, we firstly use Hampel filter to remove outliers to improve the data quality; subsequently, in order to further improve the accuracy of CSI data in the time series, we apply the Discrete Wavelet Transform (DWT) technique for noise reduction against high-frequency noises caused by fluctuations in the transmission rate or changes in the external environment, so as to effectively eliminate the target activity-independent interference signals that are not related to the target activity.

### 2.2. Human Activity Recognition with CSI

In recent years, deep learning techniques have made remarkable achievements in the fields of computer vision, natural language processing (NLP), and medical image analysis, which have inspired researchers to apply them to new explorations in the field of human activity recognition based on wireless signals. As one of the core algorithms for image recognition, Convolutional Neural Network (CNN) was first considered for processing CSI (Channel State Information) signals. Zou et al. [31] designed a Wi-Fi-based unsupervised-domain adaptive gesture recognition scheme, WiADG, which uses a CNN as a classifier to reduce the domain difference between the source and target domains, thus improving the generalisation performance of the classifier. In addition, Ma et al. [32] developed the SignFi system, which successfully recognised 276 different hand gestures based on CNNs. Although CNNs have made significant progress in the field of image processing, when dealing with dynamic signals such as CSI, CNNs relying on convolutional operations are difficult to capture the dependencies between different moments.

Recurrent Neural Networks (RNN) are able to remember the outputs of previous time steps and use them as inputs to the current time step through the recurrent structure of their hidden layers, enabling the network to capture dynamic changes in a time series. Ding et al. [33] proposed a deep recurrent neural network (HARNN) that incorporates an innovative two-layer decision tree model from a multidimensional statistical chart to extract key features and classify different human actions using Recurrent Neural Networks (RNN). However, when dealing with longer sequences, RNNs are prone to face the gradient vanishing or gradient explosion problem, which limits their ability to model long time dependencies. To solve this problem, LSTM (long short-term memory network), as an improved version of RNN, enhances the network’s ability to learn and retain long-time dependencies through the introduction of “memory units” and three gating mechanisms: input gate, forgetting gate, and output gate. Yousefi et al. [22] used the long short-term memory network (LSTM), which is a new version of RNN, in deep learning to learn and retain long-time dependencies. The long short-term memory network is used to process CSI data streams, which demonstrated significant performance improvement compared to traditional machine learning techniques. The bidirectional long short-term memory (Bi-LSTM) network proposed by Graves et al. [34] pays special attention to the interdependence between sequence elements, and by fusing the outputs of the forward and reverse LSTM units, it forms an integrated and comprehensive representation that contains both before and after time information. Lee et al. [35] used a single-layer CNN in combination with a BiLSTM to achieve efficient recognition of eight daily activities.

Although LSTM and BiLSTM partially solve the gradient vanishing problem of RNN by introducing a gating mechanism, the training time still increases significantly when dealing with large-scale data and long time series. In addition, CSI signals are not only significantly time-dependent, but also contain complex spatial features, such as multipath effects and channel fluctuations, the capture of which requires the model to be more locally aware. However, LSTM focuses mainly on time series and is relatively weak in modelling spatial features. Therefore, when dealing with complex and multi-dimensional signals like CSI, combining other models (e.g., self-attentive mechanisms) to capture global and local features more comprehensively may be a superior solution.

In 2017, Google proposed the Transformer model, which, with its core feature, the attention mechanism, effectively reveals the interdependencies between features and significantly optimises the process of information capture and delivery. Subsequently, Chen et al. [36] designed a bidirectional long- and short-term memory network (ABLSTM) by introducing an innovative attentional mechanism, taking into account both the forward and backward data streams of CSI signals, which led to more accurate human activity recognition. Zou et al. [20] designed the MetaFi++ system, which used convolution to map the channel state information to the human posture feature points, and explored the spatial information of human posture through the self-attention mechanism, achieving excellent performance.

With the wide application of the Transformer framework in image processing, the Visual Transformer (ViT) [18] was born. The innovation of ViT lies in dividing an image into a series of small image patches and transforming these patches into serialised data through linear embedding. This strategy, combined with the self-attention mechanism, enables ViT to comprehensively capture the global associations within an image, greatly enriching the depth and dimension of feature representation. ViT has not only achieved outstanding achievements in the field of natural language processing, but also opened up new research directions in the field of computer vision and pushed forward the innovative development of image analysis and processing. In this context, researchers began to try to apply ViT to the processing of CSI data, hoping to establish global correlation in the whole Wi-Fi channel signal sequence and capture the dependencies between different antenna data through ViT. Abdel-Basset et al. [37] designed a DL model—the H2HI-NET, which combines the capabilities of residual learning and Transformer to successfully extract spatial features of human activities. On the other hand, Yang et al. [21] proposed WiTransformer, which is a new pure ViT-based strategy combining an improved Transformer architecture, a joint space-time transformer (UST), and a separated Spatio-Temporal Transformer (SST), which effectively implements Wi-Fi-based human gesture recognition and shows great task robustness.

## 3. Method

This section describes in detail the processing of the UT-HAR dataset and Widar3.0 data, and the overall network structure of VBCNet.

### 3.1. Data Processing

**Figure 2:**
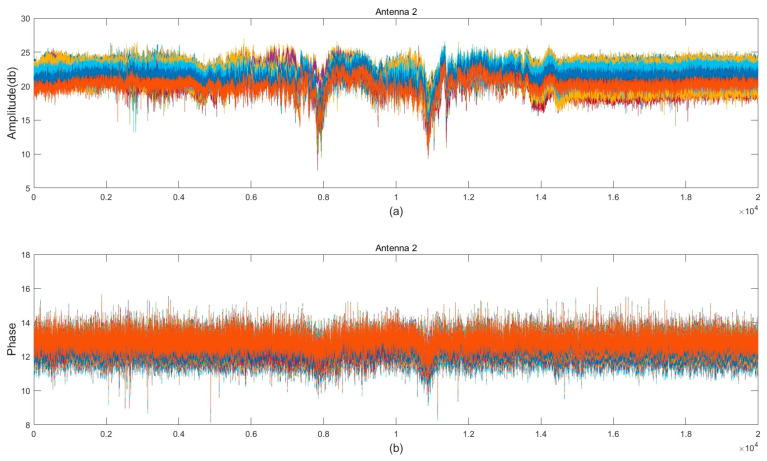
Magnitude and phase waveforms of Walk, different colours represent different carriers. (**a**) Amplitude waveform, (**b**) phase waveform.

Due to the complex surrounding environment, the collected channel state information (CSI) data are often accompanied by abundant noise. The phase component of CSI is particularly sensitive to factors such as ambient noise and unsynchronisation between hardware. In contrast, amplitude information exhibits greater resilience in the face of these disturbances, providing more stable and reliable signal characteristics. The magnitude and phase obtained for the same antenna for the same action are shown in Figure 2. In addition, parsing these signals often requires tedious and complex preprocessing work as the phase information is prone to jumps. For these reasons, we decided to mainly utilise the amplitude information of the dataset in the subsequent experiments.

**Figure 3:**
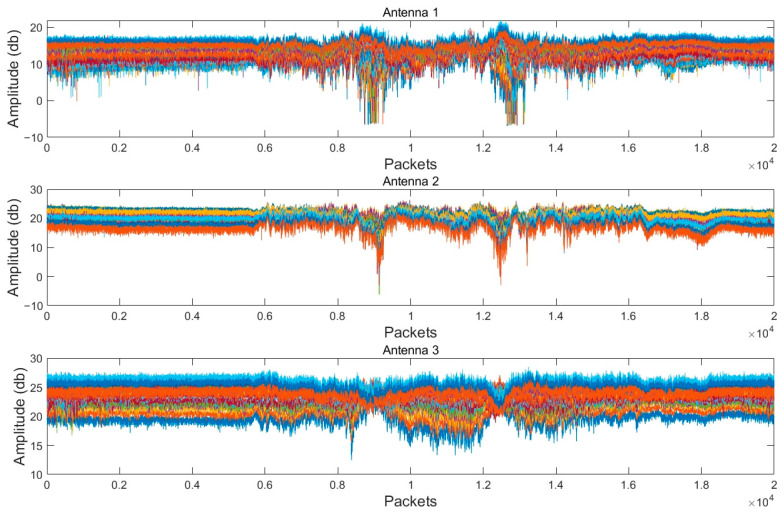
CSI amplitude waveforms of the “Walk” obtained from each of the three antennas.

The dataset UT-HAR [22] contains seven human actions: walking, running, standing, lying down, sitting, falling, and picking up, where each action is stored as a CSV file. The file contains 181 columns, with the first column recording the timestamp, columns 2 to 91 capturing the amplitude values, while columns 92 to the end record the phase information. Given that the data are captured at a frequency of 1000 Hz and a duration of 20 s, each CSV file typically contains about 20,000 rows of data. Take the action of “Walk” for example; its amplitude waveform is shown in Figure 3. It can be observed that the amplitude signal is relatively smooth when there is no movement at rest; during the movement, such as from 6 to 14 s, the amplitude waveform fluctuates significantly; and once the movement stops, the waveform returns to a flat level.

**Figure 4:**
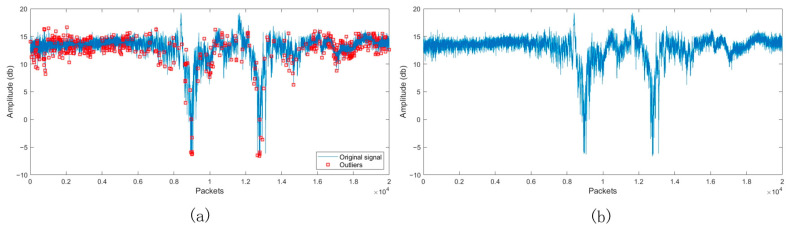
Comparison before and after outlier processing. (**a**) Original CSI Signal (**b**) CSI Signal after Hampel filtering.

Observation of Figure 3 reveals the presence of numerous outliers, which are significantly deviated from their left and right neighbours. In order to eliminate these outliers, we plan to process them using a Hampel filter. For high-frequency fluctuating signals (e.g., ECG signals), the filtering is usually set to a window size of 3~5 and a standard deviation multiplier of 3~4. For smoother signals (e.g., ambient temperature monitoring), it is usually chosen to have a larger window size of 5~7 and a standard deviation multiplier of 2.5~3. Given that the CSI data belong to the type of moderately fluctuating signals, we have decided to set the window size of 5, the standard deviation multiplier of 3, in order to balance the filtering effect and the sensitivity of the anomaly detection. Figure 4a shows the original signal with red boxes marking the anomalies, while Figure 4b shows the result of the signal after being processed by the Hampel filter and removing the outliers. The results show that the CSI waveform processed by the Hampel anomaly filter is not smoother than the original waveform, although the local interference is reduced.

**Figure 5:**
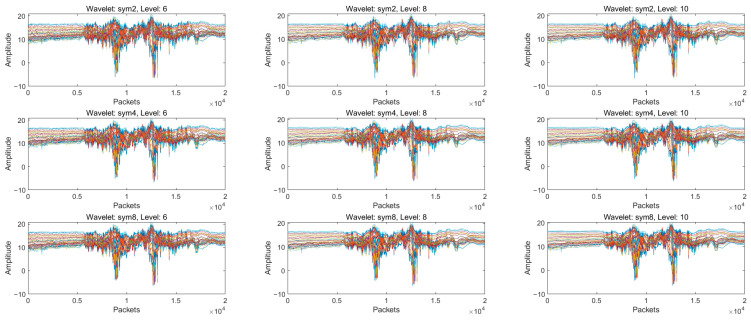
CSI signals processed with different Symlets and decomposition layers.

In order to exclude high-frequency noise unrelated to the target behaviour and to improve the accuracy of the time-series CSI data, we decided to further apply the Discrete Wavelet Transform (DWT) for noise reduction after pre-processing with a Hampel filter. In order to retain the signal details more accurately during the denoising process, we use the heuristic SURE method (least square error estimation) to automatically select the best threshold value and smooth the signal through the soft thresholding method to optimise the noise reduction effect. Meanwhile, in order to simplify the processing flow, we uniformly apply global thresholds at all wavelet decomposition levels to effectively cut the noise. In the experiments, we use 2nd, 4th, and 8th order Symlets wavelets and 6th, 8th, and 10th order wavelet decompositions to process the signals, respectively. The results show (as shown in Figure 5) that the 4th order Symlets provide a good balance between capturing signal details and avoiding excessive smoothing. At the same time, the use of 8-layer wavelet decomposition depth can meet the needs of longer signals, highlighting the key features and helping the extraction of subsequent action information. After the noise reduction process, the CSI signal shows significant noise reduction effect, and the signal characteristics are clearly displayed, which lays a good foundation for the subsequent analysis.

For CSI data collected in different environments, it usually contains a large amount of noise and the signal fluctuations show differences due to the different positions of the Wi-Fi antenna relative to the human body. These signal fluctuations cannot be converged at different locations by denoising methods. To address this problem, Zhang [23] proposed an environment-independent feature, the body-coordinate velocity profile (BVP), for such situations. Specifically, human activities have unique velocity distributions in each body part involved, and such distributions can be used as an effective characterisation of the activity. By capturing the velocity patterns generated by different body parts during various action phases, the BVP is able to quantify the velocity characteristics of a gesture, thus effectively removing the influence of the environment. In this paper, we will use the original UT-HAR dataset with noise reduction processing and the BVP data in Widar3.0 to validate the performance of the VBCNet model.

### 3.2. VBCNet Network Architecture

**Figure 6:**
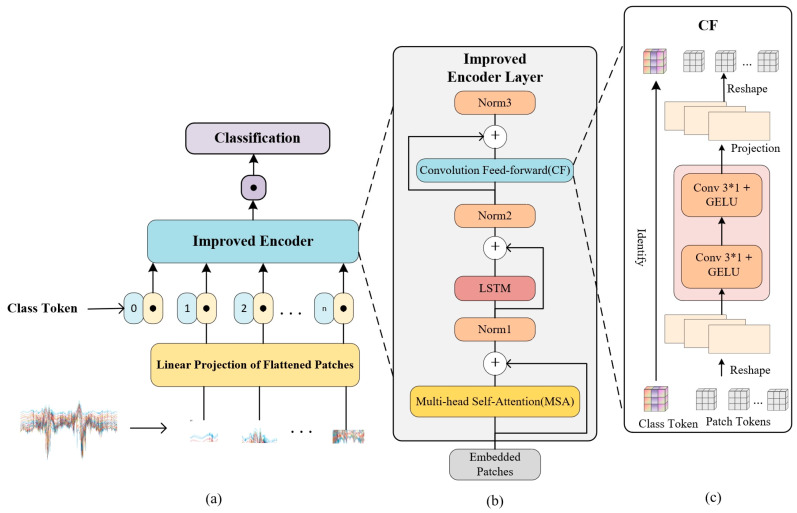
(**a**) VBCNet model structure. The input data in the figure is an example only; the input data is not a CSI data map, but a sequence of CSI data. (**b**) Improved encoder. (**c**) Convolutional Feed-forward (CF) module.

VBCNet improves the Encoder layer on the ViT model and adds the CF module, and the overall structure is shown in Figure 6.

ViT was originally designed for processing visual data and is usually applied to image classification tasks. It divides an image into a series of patches, each of which is fed into the model as a “token”. This structure allows ViT to capture both local and global patterns in an image, making it ideal for visual tasks such as object recognition and segmentation. However, ViT is also suitable for non-visual data processing, such as CSI sequences, where segments of CSI data can be input into ViT as “tokens” in the sequence for effective feature learning and processing. First, the CSI sequence is chunked similar to an image at the embedding module. The input data $x\in R^{B\times C\times H\times W}$, where B denotes the batch size, C is the number of input channels, and H and W denote the number of rows and columns of the input sequence, respectively, i.e., H×W would represent the data size of a CSI sequence of human activities. In order to process these high dimensional data, these data are divided into N∈H/P_h×W/P_w patch blocks, where P_h denotes the width of each patch block and P_w denotes the length of each patch block. At this point, the output $x\_p\in R^{B\times N\times d}$ is obtained, where d is the embedding dimension, and subsequently, an additional class token is introduced into the sequence, and the final size of the input tokens is $x\_cls\in R^{B\times (N+1)\times d}$.

#### 3.2.1. Improved Encoder Layer

The Encoder layer is the core part of ViT, which consists of a multi-head self-attention mechanism and a feed-forward neural network. Compared with traditional convolutional neural networks (CNNs), ViT is able to capture a wider range of contextual information through the self-attention mechanism, and when processing CSI signal data, ViT is able to assign higher weights to key features and extract fine-grained time-series data to some extent feature information; however, it is due to this self-attention mechanism that ViT is not capable of processing small sample data and capturing local features. To solve this problem, we change the original Encoder layer as shown in Figure 6b, and the detailed steps are as follows:

Each Encoder block contains a multi-head self-attention layer, a convolutional feed-forward network (CF), and residual connections. First, the output sequence $x\_cls\in R^{B\times (N+1)\times d}$ of the position embedding layer is fed to the multi-head self-attention mechanism, which maps the input into three matrices representing the query (Q), the key (K), and the value (V), which have dimensions $q,k,v\in R^{B\times h\times (N+1)\times d\_h}$, where h is the number of the attention heads and d_h=d/h denotes the dimension of each attention head. Next, the attention weights are calculated using these three matrices and the attention score is calculated as:

(4) $$Attention\left(Q,K,V\right)=softmax\left(\frac{QK^{T}}{\sqrt{d_{k}}}\right)V,$$

where $QK^{T}$ is the dot product computation, denoting the use of a query vector Q to match or measure the correlation with each key K; where $\sqrt{d_{k}}$ is a scaling factor to prevent the dot product of high-dimensional vectors from leading to gradient explosions; and where $d_{k}$ is the dimensionality of the keys or query vectors, which is usually equal to the dimensionality of each attention header, d_h. This factor ensures that the dot product results are smoother in the high-dimensional case to avoid the gradient being too large or too small. Multi-head attention, i.e., multiple pairs of Q, K, and V matrices are generated, multiple independent attention weight calculations are performed, and finally the resulting individual attention weight matrices are spliced together and a single linear transformation is applied to them as output. Subsequently, the results are subjected to a dropout and linear projection to remap the output of the multiple attention back to the original embedding dimension, i.e., $x\_attn\in R^{B\times (N+1)\times d}$, followed by a residual join. Considering that CSI (channel state information) sequences have obvious temporal characteristics, we introduce a layer of LSTM network in order to enhance the model’s ability to extract temporal features. Since LSTM may suffer from gradient vanishing when dealing with long sequences, we enhance the stability of the model by residual joining and layer normalisation to ensure that the gradient does not vanish during the training process. In the subsequent ablation experiments, we will compare the effects of normal LSTM and BiLSTM on the network performance. The number of hidden units of the LSTM is h_lstm, and the output dimension of each patch is h_lstm. The output of the LSTM layer is denoted as $x\_lstm\in R^{B\times (N+1)\times h\_lstm}$, and in order to match the embedding dimension d, the output is mapped back to $x\_lstm\_proj\in R^{B\times (N+1)\times d}$ through a fully connected layer, followed by residual concatenation and normalisation to obtain the data $x\_norm\in R^{B\times (N+1)\times d}$.

#### 3.2.2. CF Module

In ViT, a feed-forward network (FFN) is often used to enhance the feature representation capability. The FFN improves the representation capability of the model by introducing complex nonlinear relationships, but because it consists of two linear layers and an activation function it is unable to effectively capture the local features in the input data, especially when dealing with sequential data, and it cannot make full use of the spatial or temporal structural information of the data. For this reason, this study proposes to replace the original feed-forward network with a convolutional feed-forward (CF) module, as shown in Figure 6c. The CF module enables the model to better capture the local structure in the input sequences by introducing convolutional operations, which enhances the learning ability of neighbouring features. In addition, the use of convolutional kernels of different sizes enables the simultaneous extraction of features at multiple scales, leading to a more comprehensive understanding of the multilevel information of the input data. By splicing the outputs of different convolutional kernels, the complexity and expressive power of the model is enhanced, which helps to capture more feature information.

In the CF module, the input normalised data $x\_norm\in R^{B\times (N+1)\times d}$ is first rearranged into $x\in R^{B\times d\times (N+1)}$ in order to facilitate the 1D convolution operation. Next, two convolution kernels of size 3×1 are used to perform convolution operations on the sequence to capture local relationships between neighbouring elements and extract local features. In our experiments, we will also explore the effects of different number of convolutional layers and convolutional kernel sizes on the model performance. The GELU activation function is used to enable the model to learn more complex features, and then the outputs of the convolutional layers conv1 and conv2 are spliced in the channel dimension, combining features from different convolutional kernels to enhance the diversity of feature representations, obtaining $x\_ff\in R^{B\times 2\times d\times (N+1)}$, which is then projected by 1D convolution to preserve the useful information, and the spliced high-dimensional features are mapped back to the input dimensions, and the final output is rearranged as $x\_ff\_proj\in R^{B\times (N+1)\times d}$ and regularised by dropout operation before output.

We residually concatenate the output of the CF module with its inputs and use it as the improved Encoder layer output after one normalisation. Finally, the sequence features are aggregated into a fixed-size feature vector $x\_mean\in R^{B\times d}$ by global average pooling, which is then mapped into classification scores by a linear layer, and the final output for classification is the vector result $x\_classifier\in R^{B\times classes}$.

## 4. Experiments

In order to fully evaluate the advantages of the VBCNet model, we first compare it with several CSI-based action recognition models and popular deep learning methods on two datasets, UT-HAR [22] and Widar3.0 [23]. The benchmark methods compared include traditional LSTMs, Convolutional Neural Networks (CNNs), as well as more advanced self-attention mechanisms and hybrid network architectures. Through these comparisons, we aim to demonstrate the unique advantages of VBCNet in spatial and temporal feature extraction, and to validate its generalisation ability on different datasets. Next, in order to explore the design principles and performance of VBCNet in more depth, we conduct an ablation study on the UT-HAR dataset. Specifically, we designed a series of comparison experiments by adding the LSTM layer and the convolutional feedforward (CF) module to the network separately, aiming to analyse the contribution of each module to the model performance. Through these experiments, we not only validate the effectiveness of the overall architecture of VBCNet, but also reveal the specific roles of each module in spatial and temporal feature extraction. This ablation study helps us to understand more clearly the excellent performance of VBCNet in handling complex action recognition tasks. In addition, during model training, we used an NVIDIA RTX3060 GPU, set the learning rate to 0.001, used CrossEntropyLoss as the loss function and optimised it using Adam op-timiser, and trained with a batch size of 64.

### 4.1. Comparison with Other Models

To evaluate the performance of the VBCNet model, it was compared with several benchmark methods and popular models based on CSI action recognition. In the literature [22], the UT-HAR dataset was first proposed and the LSTM model and HMM model, which are widely used in deep learning, were employed for activity classification. Wang et al. [38] developed a sparse self-encoder (SAE) network capable of recognising position, activity, and gesture at the same time. Chen [36] introduced a BiLSTM model employing an attention mechanism (ABLSTM), which achieved significant improvement in action recognition performance. Tang [39], on the other hand, designed a network structure with CNN and GRU in parallel to effectively cope with the complexity of signal propagation and the diversity of motion amplitude, and achieved high-accuracy recognition of human behaviours. Sheng et al. [15] designed an end-to-end deep learning framework that uses CNN features as the two-layer inputs to a bidirectional LSTM model in an attempt to simultaneously mine spatial and temporal information.

#### 4.1.1. Validation of the UT-HAR Dataset

The results of testing VBCNet with other models on the UT-HAR dataset are shown in Table 1.

**Figure 7:**
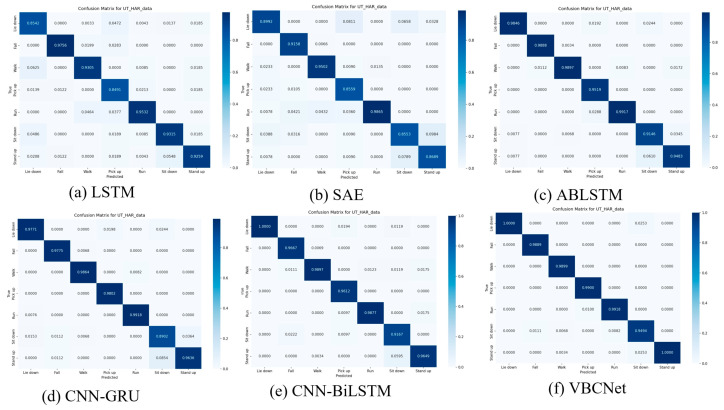
Confusion matrix for six models on the UT-HAR dataset.

To better illustrate the performance of the VBCNet models in terms of spatio-temporal feature extraction, we plotted the detailed confusion matrices of the six models on the UT-HAR dataset, as shown in Figure 7.

In a series of models using the UT-HAR dataset, the accuracy of the traditional LSTM model was 91.95%. For both “Sit down” and “Stand up” behaviours, the probability of LSTM misclassifying one behaviour as the other is significantly lower than that of the SAE model (5.48% probability of LSTM misclassifying “Sit down” as “Stand up”). The probability of LSTM misjudging one behaviour as another is significantly lower than that of the SAE model (LSTM predicts “Sit down” with a 5.48% probability of misjudging it as “Stand up”, while SAE predicts it with a 7.89% probability). However, for dynamic behaviours such as “Lie down” and “Pick up”, the misclassification phenomenon of LSTM is more prominent, which indicates that although LSTM has the advantage of processing time series data, it is still insufficient in dealing with the task of identifying complex behaviours. This indicates that although LSTM has advantages in processing time series data, it is still insufficient in dealing with complex behaviour recognition.

The overall accuracy of the SAE model is 92.61%, and the misclassification phenomenon is more serious in the categories of “picking up” and “sitting down”, with accuracy rates of 85.59% and 85.53%, respectively. This indicates that SAE has certain limitations in processing high-dimensional, time-rich relational data, and it is difficult to accurately capture the subtle differences between actions.

The BiLSTM with the introduction of the attention (ABLSTM) mechanism significantly improves the classification performance with an accuracy of 97.66%. In particular, the excellent performance in the recognition of “Sit down” and “Stand up” actions demonstrates that the attention mechanism can help BiLSTM better capture the details of the actions, thus improving the classification accuracy. In addition, the attention mechanism enhances the recognition of dynamic behaviours. For example, the accuracy of the “Pick up” category reaches 95.19%, which is significantly higher than that of LSTM and SAE, demonstrating its advantages in capturing temporal and movement details.

Combining the spatial feature extraction of CNN and the temporal feature capture of GRU, the CNN-GRU model achieves an accuracy of 97.52%. It performs well in classifying complex dynamic behaviours (e.g., “Run” and “Pick up”), but performs less well in distinguishing between “Sit down” and “Stand up”, with accuracy rates of 95.7% and 95.7%, respectively. “Stand up” had accuracies of 89.02% and 96.36%, respectively, which indicates that there is still room for improvement in the temporal feature extraction of certain categories.

CNN-BiLSTM performs close to ABLSTM by integrating the extraction of spatial and temporal information, and especially excels in dynamic behaviours. The prediction accuracies of “Run” and “Pick up” are 98.77% and 96.12%, respectively, demonstrating the model’s high sensitivity in short-time sequence action recognition.

VBCNet demonstrates excellent performance in human activity recognition tasks, with its innovative structural design improving the accuracy of action recognition to 98.65%. VBCNet consistently demonstrates optimal or near-optimal performance in classification tasks across a wide range of behavioural categories, and its performance on specific categories in particular provides important observations and insights for research. For example, in the category of “Lie down”, since both ”Lie down” and “Pick up” have similar downward movement characteristics, coupled with their temporal features being less recognisable, the local feature extraction capability of the model is highly required. Models with strong local feature extraction ability (e.g., CNN-GRU, CNN-BiLSTM, and VBCNet) significantly outperform the LSTM and SAE models in this category, with the lowest misclassification rate in VBCNet. This suggests that the introduction of the ViT module in VBCNet effectively enhances the model’s ability to capture long-range dependent features. In addition, VBCNet achieves the best classification accuracy on the most confusing actions of “Sit down” and “Stand up”. This further validates that the introduction of the convolutional feedback (CF) module and the combination of BiLSTM in ViT can effectively improve the model’s ability to extract local and temporal features and significantly enhance the overall classification performance.

These results show that different models have their own strengths when dealing with specific tasks: the CNN model excels in local feature extraction and is suitable for tasks such as gesture recognition that focus on the details of local actions, while the LSTM model excels in dealing with long-distance dependencies and is suitable for dynamic actions that require contextual understanding, such as running and jumping. However, LSTM-only models are limited in their performance in the absence of local feature support and are usually used in combination with CNNs. ViT models are excellent in global feature modelling, and VBCNet, which combines CNNs and LSTMs, has the unique advantage of combining global dependencies with local feature mining.

**Figure 8:**
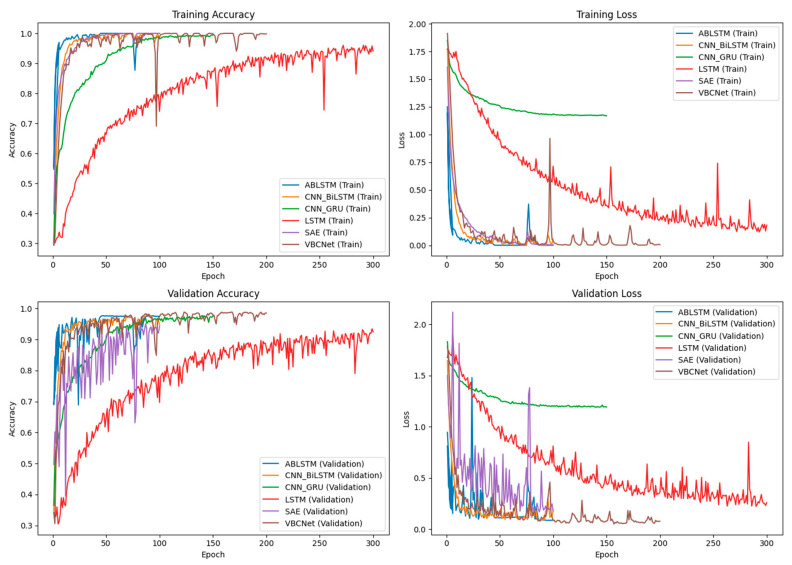
Training curves for the six models.

The training curves for several models are shown in Figure 8.

As can be seen in Figure 8, the accuracy of ABLSTM rises the fastest during the training process, rapidly approaching the maximum accuracy and stabilising within the first 50 epochs. This is closely followed by the CNN_BiLSTM, SAE, and VBCNet models, which reach a steady state after about 100 epochs. In contrast, CNN_GRU converges slowly and gradually stabilises after about 150 epochs. Meanwhile, the loss value of CNN_GRU on the training set is significantly higher than that of other models, and even though we optimise it by increasing the code complexity and adjusting the loss function, we still fail to make its loss value reach a similar level as that of other models. On the test set, the loss value of CNN_GRU also stays at a high level, slightly higher than its loss value on the training set. However, it is worth noting that the accuracy of CNN_GRU remains high on both the training and test sets, approaching models such as ABLSTM. We speculate that this is because the output probability distribution of the CNN_GRU model may not be as stable or as well calibrated as ABLSTM and SAE. Despite the close accuracy, the uncalibrated model tends to output a more dispersed probability distribution with lower confidence in each correctly predicted category, which may have contributed to its higher loss values. In addition, the LSTM model had the slowest decline in loss during training and had significantly lower training and testing accuracies than the other models. This suggests that for complex CSI data, the LSTM structure may be less effective in extracting useful features. Overall, our VBCNet model performs well in terms of accuracy and, at the same time, performs well in terms of convergence speed, showing its advantages in processing CSI data.

To ensure the reliability of the results, we introduced statistical significance tests to verify the validity of the models. Given the need to compare the performance of multiple models, we used ANOVA for the analysis and the results are shown in Table 2. Table 2 shows that the average accuracy of VBCNet is 6–7% higher than other models such as LSTM and SAE. Analysed by *t*-test, the *p*-values of both LSTM and SAE are less than 0.001, indicating that this performance improvement is highly statistically significant. For ABLSTM, CNN-GRU, and CNN-BiLSTM, although the performance gap is relatively small, the *p*-values are all less than 0.05, which still show significant differences. These results further demonstrate that the improvement of VBCNet is statistically significant.

#### 4.1.2. Validation of the Widar3.0 Dataset

To validate the robustness of the model, we further evaluated VBCNet on the Widar3.0 dataset, which covers 3 different environments, 16 users, 22 gestures with different positions and orientations, and the BVP data of these gestures, and this diversity poses a new challenge for gesture recognition. The advantages of VBCNet in spatial and temporal feature extraction are further highlighted by the experimental results of VBCNet with other models on this dataset. Due to the large variation in the number of samples for different gestures, we have analysed the model in detail in terms of both accuracy and recall dimensions.

The accuracy and training time of several models on the Widar3.0 dataset are shown in Table 3.

As can be seen in Table 3, for the BVP data in the cross-domain Widar3.0 dataset, VBCNet performs the best with an accuracy of 77.92%, which is significantly better than the other models. It is followed by CNN-BiLSTM with an accuracy of 71.40%, while LSTM performs the worst with 62.39%. In terms of training time, VBCNet also performs well, taking only 7755.91 s to train all samples, making it the model with the shortest training time. This is due to the efficient extraction of features by the convolutional module and self-attention mechanism in VBCNet, which significantly reduces the training time. In comparison, the training time of both LSTM and CNN-GRU exceeds 7900 s. In addition, VBCNet also has an advantage in testing time, with an average processing time of only 2.53 ms per test sample, which is slightly shorter than the other models. This indicates that VBCNet not only performs well in accuracy and training efficiency, but also has high inference efficiency in practical applications.

**Figure 9:**
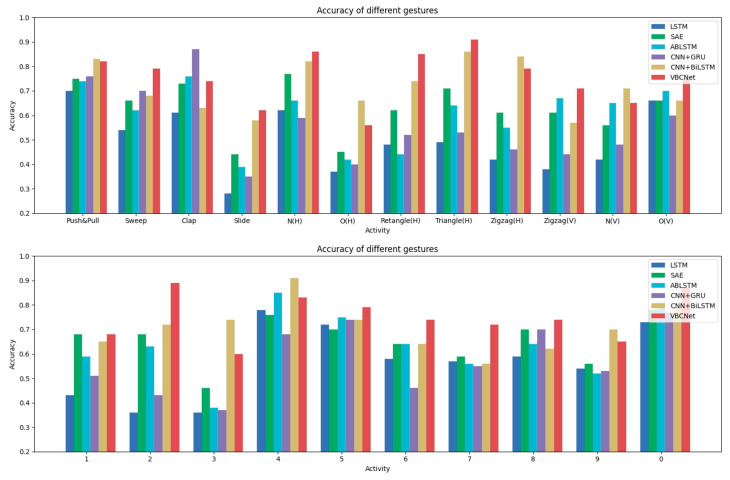
Accuracy of different models for 22 gestures on Widar 3.0 dataset. (H) represents Horizontal and (V) represents Vertical.

The specific recognition of the 22 gestures is shown in Figure 9.

**Figure 10:**
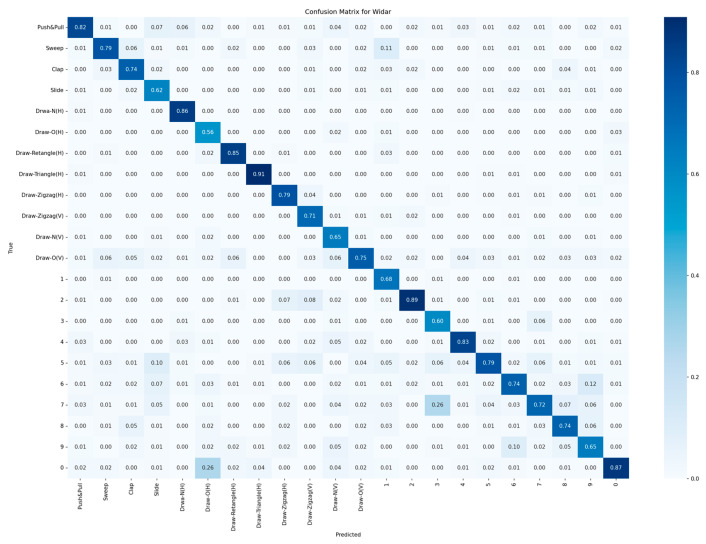
VBCNet confusion matrix on 22 gestures.

It can be found that among the six models, the VBCNet model achieves the optimal accuracy rate for 13 gestures, among which there are several gestures with lower accuracy rates—“Slide”, “Draw-O(H)”, and “Draw-3”. When collecting the “Slide” action, the behaviour of the limb is blocked by the torso, and the reflection of the Wi-Fi signal is not strong compared to other actions; in addition, this action is relatively simple and featureless compared to other gestures, so the model is unable to extract effective features for it. For “Draw-O(H)”, this action is similar to “0”, and it is difficult to reflect the key features through the BVP data, so the model has a high probability of classifying it as “0” when predicting it (the confusion matrix of VBCNet is shown in Figure 10). As for the gesture “Draw-3”, it is mostly predicted as the gesture “Draw-7”. The main reason is that the digit “3” and the stroke structure of the digit “7” are close to each other in some writing styles, especially when the lower part of the digit “3” is elongated or the upper horizontal and diagonal lines of the digit “7” are not obvious, the outlines of the two are similar, increasing the possibility of confusion. The similarity of the two outlines increases the likelihood of confusion.

**Figure 11:**
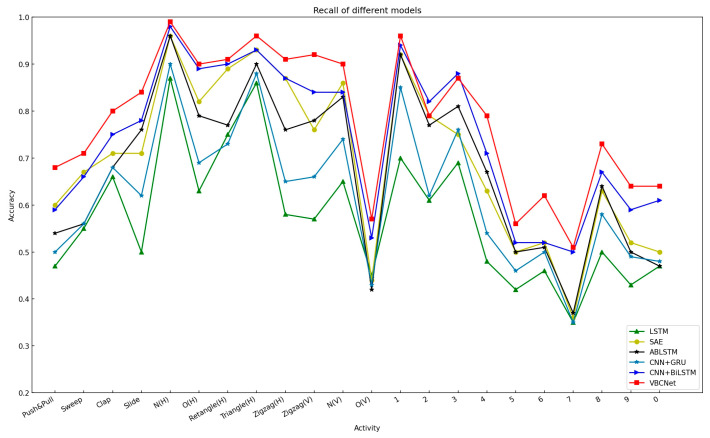
Recall of 22 gestures.

The recall of the model for each gesture is shown in Figure 11. The relatively high recall of VBCNet for all gestures indicates that it has stronger generalisation ability in gesture recognition, especially for gestures with fewer samples (e.g., numeric gestures 0–9) where it still performs well. However, for the “Slide” and “Draw-3” gestures, the recall remains high although the accuracy is low. This suggests that other gestures are rarely misclassified as these two gestures, reflecting the fact that the features of these gestures are not significant enough for the model to extract them effectively. In contrast, the gestures “Draw-O(V)” and “Draw-7” have lower recall but higher accuracy, which suggests that the model, although unable to capture enough samples in the positive category, seldom misclassifies samples from other categories when classifying them as these two categories. This means that the model is relatively “cautious” in handling these gestures and would rather miss than misclassify them.

### 4.2. Ablation Study

To further explore the excellence of VBCNet in spatial and temporal feature extraction, we gradually introduce the LSTM layer and the CF module into the network and design a series of comparative experiments to analyse in detail their impact on the model performance. In the experiments, we try unidirectional and bidirectional structures on the LSTM layer to investigate how different directionality of the LSTM affects the ability of temporal feature capture. At the same time, we adjusted the size of the convolution kernel and the number of layers of the convolutional layers on the CF module to analyse the impact of changes in the convolutional parameters on spatial feature extraction.

#### 4.2.1. LSTM Layer on the Model

For some specific movements, such as Lie down and Sit down, both involve a similar squatting motion at the beginning of the activity. However, the key difference between the two is that “Lie down” is followed by a squat that is further followed by a lie-flat movement, i.e., a full extension of the body. This subtle spatial difference requires a strong spatial feature extraction capability, and the Visual Transformer (ViT) demonstrates a significant advantage in capturing the spatial difference between the different movements. In addition, a spatial feature module such as ViT can detect subtle features in the gestural changes of movements, thus accurately classifying similar movements.

**Figure 12:**
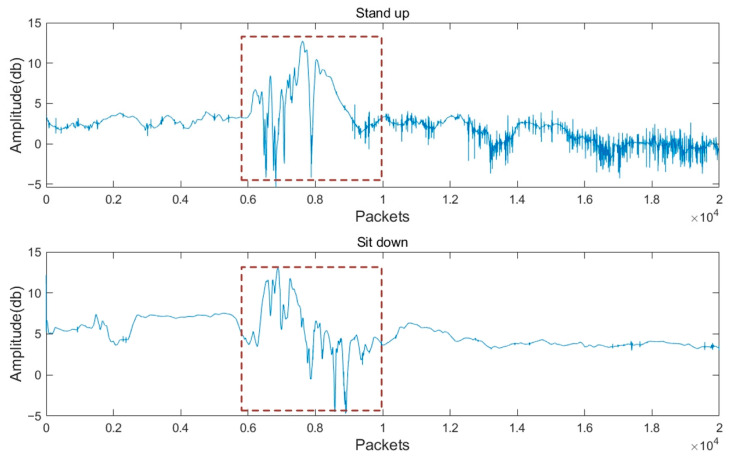
Signal representation of “Stand up” and “Sit down” actions. The dashed frame shows the signal fluctuations when these two actions occur.

Further, for the two behaviours “Stand up” and “Sit down”, which can be viewed as reversing each other from a certain point of view, there is a high degree of similarity in features, and it is more reasonable to deal with this kind of time-series features. LSTM is more reasonable to deal with this kind of time series because it is good at capturing the temporal dependency in the sequence. The signal representation of the two actions is shown in Figure 12.

It may be difficult to fully capture the interaction and integration of spatial and temporal features of these behaviours if we rely only on ViT or traditional LSTM to deal with the complex dynamic characteristics of these behaviours independently. ViT is excellent in capturing spatial features but deficient in dealing with sequential dependencies; comparatively, LSTM has an advantage in modelling the dependencies of time-series data, but is slightly deficient in extracting spatial features. Therefore, in order to comprehensively improve the performance of the model, we introduced the LSTM layer on top of ViT and constructed the VBCNet model. In this way, the ViT module can deeply explore the complex spatial structure in CSI sequences, while LSTM can effectively capture the dynamic changes in behaviours through time-dependent modelling and realise the efficient fusion of spatial and temporal features, so as to achieve higher accuracy in the recognition of complex behaviours. In order to further investigate the difference between LSTM and BiLSTM in behaviour recognition, we designed a series of combined experiments, combining unidirectional and BiLSTM with ViT, respectively, to analyse the effect of different structures on feature extraction. The experimental results are shown in Table 4.

According to the experimental results in Table 4, it can be learnt that the recognition accuracy of the seven actions using the ViT network model alone is 96.38%. In comparison, the accuracies of LSTM and BiLSTM are 92.61% and 93.25%, respectively. This suggests that although LSTM and BiLSTM perform well in temporal feature extraction, relying only on temporal features may not yield high accuracy when dealing with datasets with high-dimensional spatial and temporal features.

ViT networks are able to mine complex spatial features effectively, which is crucial for improving recognition accuracy. However, when the dataset contains rich temporal features at the same time, ViT alone may not be able to fully utilise the temporal information. By adding an LSTM layer to ViT, we observe that the recognition accuracy improves to 97.05%, and the accuracy is even higher with the addition of a BiLSTM layer, reaching 97.66%, which indicates that the ability to combine spatial and temporal features significantly enhances the overall performance of the model for CSI sequence learning.

#### 4.2.2. Impact of CF Module

The CF module is gradually introduced into the hybrid network model of ViT, LSTM, and BiLSTM for validation, while keeping other parameters constant. Table 5 lists the experimental results with different modifications.

As can be seen from the results in Table 5, after the introduction of the Convolutional Feed Forward (CF) module in the ViT network, the accuracy is significantly improved to 96.85%, which verifies the effectiveness of the CF module in enhancing the ability of ViT to acquire local and multi-scale information. Further, after adding LSTM to the Encoder layer, the accuracy rate is improved to 98.34%, indicating that the introduction of LSTM in time series analysis can significantly enhance the classification performance. Ultimately, after using BiLSTM instead of LSTM, the accuracy is further improved to 98.65%, demonstrating the advantages of bidirectional LSTM in capturing time-series features, which further improves the performance of the model in subtle behaviour recognition.

To explore the impact of convolutional layer configurations on experimental outcomes, we performed comparative experiments using single, double, and triple convolutional structures. Additionally, we evaluated the effects of varying convolutional kernel sizes. The experimental results are shown in Table 6.

From the experimental results in Table 6, it can be seen that when the CF module uses single-layer convolution, the highest accuracy of 98.05% is achieved when the convolution kernel size is 5. When three layers of convolution are used, the accuracy further increases to 98.33%, which is slightly higher than that of single layer convolution. This suggests that increasing the number of convolutional layers has some improvement in the performance of the model, but its improvement is limited.

However, when using a two-layer convolution with both convolution kernel sizes of 3, VBCNet shows the best results with an accuracy of 98.65%, which was the highest of all the experiments. This suggests that the use of smaller convolutional kernels (3) and a moderate number of convolutional layers (2) provides optimal performance in the subtle behaviour recognition task in this particular experimental configuration. This result may be explained by the fact that smaller convolutional kernels can better capture the local details of the input data, whereas too many convolutional layers may lead to overfitting or loss of information.

Comparatively, larger convolutional kernels (e.g., 7) fail to deliver better results in deeper convolutional layers, but instead may affect the performance by making training difficult or increasing the complexity of the model due to an excessive number of parameters. In particular, when using three layers of convolution, although the accuracy improved, it did not exceed the combination of a two-layer convolution plus a small convolutional kernel.

These results suggest that appropriately reducing the number of convolutional layers and using a smaller convolutional kernel instead results in better performance for a given task. This phenomenon may be related to the characteristics of fine-grained action recognition, where fine-grained spatial information is more important in the task of classifying human action recognition based on CSI data, and too many convolutional layers and a larger convolutional kernel may not be conducive to capturing these details.

## 5. Conclusions

In this paper, we propose a deep learning-based hybrid model VBCNet for processing CSI data and classifying human dynamic behaviours. VBCNet combines the advantages of ViT in deep feature extraction and the power of LSTM in time series analysis to achieve efficient behaviour classification by extracting high-dimensional feature vectors and transforming them into time series features. In addition, VBCNet replaces the feedforward network in ViT with a CF module, which enhances ViT’s ability to capture local and multi-scale information. We conducted experiments on the UT-HAR dataset and the Widar3.0 BVP dataset and compared VBCNet with traditional deep learning models (e.g., LSTM, SAE) as well as a variety of combinatorial models (e.g., ABLSTM, a BiLSTM model employing an attentional mechanism, a CNN-GRU structure in which CNNs are connected in parallel with GRUs, and a variant of CNN and BiLSTM CNN-BiLSTM) were compared. The experimental results demonstrate the superiority and robustness of the VBCNet model in classification tasks. In addition, the introduction of a CF module and a layer of BiLSTM is justified by ablation experiments.

Although VBCNet outperforms other hybrid models on both datasets, it still suffers from limitations similar to those of other models. For example, its ability to generalise data across domains in different environments is insufficient, and it tends to confuse similar actions or fails to effectively extract actions with less distinctive features. Future research should explore how to effectively reduce redundant information in CSI data, especially considering the differences in the amplitude of CSI signal fluctuations due to limb movements in different environments, and the large amount of data that may be required to process multiple activities if the environmental effects cannot be effectively mitigated, thus significantly increasing the complexity of the model. In addition, refs. [40,41] critically analysed the data, which inspired us a lot. The focus of this study is on the improvement of model architecture and performance optimisation, and we also regard data-related issues as an important research direction in the future and plan to explore them in depth in our follow-up work in order to improve the data partitioning strategy and experimental design, so as to enhance the model’s generalisation ability and the reliability of the results.

## Figures and Tables

**Table 1:** Classification results of different classification algorithms.

Classification Algorithm	Behaviours							Average (%)
	Lie Down	Fall	Walk	Pick Up	Run	Sit Down	Stand Up	
LSTM	85.4	97.6	93.1	84.9	95.3	93.2	92.6	91.95
SAE	89.9	91.6	95.0	85.6	98.6	85.5	86.9	92.61
ABLSTM	98.5	98.9	99.0	95.2	99.2	91.5	94.8	97.66
CNN-GRU	97.7	97.8	98.6	98.0	99.1	89.0	96.4	97.52
CNN-BiLSTM	100	96.7	99.0	96.1	98.8	91.7	96.5	97.80
VBCNet	100	98.9	99.0	99.0	99.2	94.9	100	98.65

**Table 2:** Analysis of ANOVA significance test.

Classification Algorithm	Average Accuracy (%)	Standard Deviation	*p*-Value with VBCNet
LSTM	91.51	0.7027	<0.001
SAE	92.50	0.7079	<0.001
ABLSTM	97.68	0.2746	0.003
CNN-GRU	97.35	0.4495	0.002
CNN-BiLSTM	97.62	0.4685	0.012
VBCNet	98.49	0.2679	-

**Table 3:** Accuracy and training time on the Widar3.0 dataset.

Classification Algorithm	Average (%)	Training Time (s) of All Training Samples	Testing Time (ms) of Each Testing Sample
LSTM	62.39	7902.49	2.58
SAE	67.80	7833.10	2.57
ABLSTM	67.15	7839.88	2.56
CNN-GRU	64.13	7984.52	2.63
CNN-BiLSTM	71.40	8024.32	2.66
VBCNet	77.92	7755.91	2.53

**Table 4:** Impact of LSTM layers on the experiment.

Molel	Accuracy (%)
ViT	96.38
LSTM	92.61
BiLSTM	93.25
ViT + LSTM Layer	97.05
ViT + BiLSTM Layer	97.66

**Table 5:** Experimental results of ablation of LSTM layer, CF module.

Model Structure	CF	LSTM	BiLSTm	Accuracy (%)
ViT	-	-	-	96.38
ViT + CF	√	-	-	96.85
ViT + CF + LSTM	√	√	-	98.34
ViT + CF + BiLSTM	√	-	√	98.65

‘√’ indicates that it has a modified network layer, ‘-‘ indicates that it does not contain this network layer.

**Table 6:** Impact of different sizes of CF modules on results.

First Convolutional Layer Kernel_Size	Second Convolutional Layer Kernel_Size	Third Convolutional Layer Kernel_Size	Accuracy (%)
3	5	7	98.33
3	-	-	97.44
-	5	-	98.05
-	-	7	97.85
3	5	-	97.87
3	-	7	97.74
-	5	7	98.40
**3**	**3**	**-**	**98.65**
5	5	-	98.25
-	7	7	98.07
3	3	3	97.33
5	5	5	97.85
7	7	7	97.58

Bolded lines are optimal configurations and optimal results.

## Data Availability

Two datasets were used in this study. The UT-HAR dataset can be found at https://github.com/ermongroup/Wifi_Activity_Recognition, accessed on 20 October 2024; the Widar3.0 dataset can be found at https://ieee-dataport.org/open-access/widar-30-wifi-based-activity-recognition-dataset, accessed on 20 October 2024; Both datasets are available at https://data.mendeley.com/datasets/dzvgyxkx2f/1, accessed on 20 October 2024, and the model section is at https://github.com/1999dzy/HAR_VBCNet, accessed on 25 October 2024.
